# Voltammetric Determination of Anethole on La_2_O_3_/CPE and BDDE

**DOI:** 10.1155/2018/2158407

**Published:** 2018-02-20

**Authors:** Mateusz Kowalcze, Jan Wyrwa, Małgorzata Dziubaniuk, Małgorzata Jakubowska

**Affiliations:** AGH University of Science and Technology, Faculty of Materials Science and Ceramics, Mickiewicza 30, 30-059 Kraków, Poland

## Abstract

In this work, DPV determination of anethole was presented using various carbon, two-diameter (1.5 and 3 mm) electrodes, that is, BDD, GC, CP, and CP doped by La_2_O_3_ and CeO_2_ nanoparticles. La_2_O_3_/CPE to our best knowledge was proposed first time. Cyclic voltammograms confirmed totally irreversible electrode electrooxidation process, controlled by diffusion, in which two electrons take part. The most satisfactory sensitivity 0.885 ± 0.016 *µ*A/mg L^−1^ in 0.1 mol L^−1^ acetate buffer was obtained for La_2_O_3_/CPE with the correlation coefficient *r* of 0.9993, while for BDDE it was 0.135 ± 0.003 *µ*A/mg L^−1^ with *r* of 0.9990. The lowest detection limit of 0.004 mg L^−1^ was reached on La_2_O_3_/CPE (3 mm), what may be compared with the most sensitive conjugate methods, but in the proposed approach, no sample preparation and analyte separation was needed. Anethole was successfully determined in specially prepared ethanol extracts of herbal mixtures of various compositions, which imitated real products. The proposed procedure was verified in analysis of commercial products, that is, anise essential oil, which contains a large concentration of anethole, and in alcohol drinks like Metaxa, Ouzo, and Rakija, in which the considered analyte occurs on trace levels. Structure and properties of the considered nanopowders and graphite pastes were investigated by EDX, SEM, and EIS.

## 1. Introduction

### 1.1. Anethole: Properties, Application, and Methods of Determination

Anethole—anise camphor, *para*-methoxyphenylpropene, CAS number: 104-46-1—is a colorless, crystalline aromatic terpenoid analogue with a characteristic sweet taste and pleasant aroma, occurring in many essential oils obtained from plants belonging to the family *Apiaceae*, such as anise (*Pimpinella anisum*), star anise (*Illicium verum*), fennel (*Foeniculum vulgare*), liquorice (*Glycyrrhiza glabra*), and caraway (*Carum carvi*) [1–4] (Table 1). It occurs naturally in the form of two isomers: *trans*- (CAS number: 4180-23-8) and *cis*- (CAS number: 25679-28-1), where naturally the *trans*-isomer is much more common [4–7].

**Figure 1:**
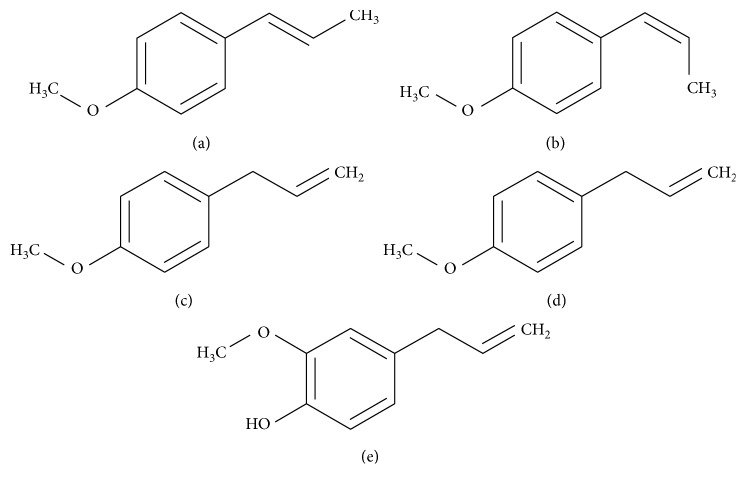
Structural formulas: (a) *trans*-anethole and (b) *cis*-anethole, and their analogues (c) estragole and (d) eugenol.

Due to its organoleptic properties, pleasant aroma and sweet taste, essential oils containing anethole have been used for centuries in the perfume, pharmaceutical, and spirit industries [1–6, 11, 12]. In pharmaceutical applications, anethole properties such as oestrogenic action, depressive action to the central nervous system, psycholeptic, insecticide, bactericidal, anticarcinogenic, anti-inflammatory, and anesthetics activity [1–6, 11, 12] are very important. The bactericidal properties of anethole are, due to lack of free phenolic group, weaker than their natural analogue—eugenol (Figure 1) [6]. In the spirit industry, anethole is present in various types of alcoholic beverages based on anise, fennel, or licorice—mainly Absinthe, Pastise, Ouzo, Rakija, and Metaxa [6, 12]. In addition, some alcohols must contain exactly the specified amount of anethole; for example, Pastise contains 1.5–2.0 g L^−1^ of this compound [12]. So, accurate determination of anethole content is one of the important stages of drinks production.

Among the quantitative methods of anethole assays in various matrices (Table 2), chromatographic techniques are dominant. Voltammetric techniques are used to evaluate the antioxidant properties of the compounds containing anethole [13] and may be useful in the classification of alcoholic beverages [14]; therefore, developing the method of anethole determination seems to be justified and interesting.

In this work, we present the possibility of *trans*-anethole determination by differential pulse voltammetry (DPV) technique. Various carbon electrodes, that is, glassy carbon electrode (GCE), boron-doped diamond electrode (BDDE), carbon paste electrode (CPE), carbon paste electrode doped by cerium(IV) oxide (CeO_2_/CPE), and carbon paste electrode doped by lanthanum(III) oxide (La_2_O_3_/CPE), were used and tested. After designation of the analytical parameters of the method and optimization, quantitative and qualitative assays of anethole were applied in four specially prepared herbal matrices similar to anethole-containing beverages and in various commercially available products of natural origin.

The obtained results are very promising and can be used in determination of anethole in a variety of matrices without analyte separation or sample preparation.

## 2. Experimental

### 2.1. Measuring Apparatus and Software

A multipurpose Electrochemical Analyzer M161 with the electrode stand M164 (both MTM-ANKO, Poland) was used for all voltammetric measurements. The classical three-electrode quartz cell of 10 mL volume was applied. Various carbon sensors were utilized as the working electrodes, that is, glassy carbon electrode (BASi, *ϕ* = 3 mm and home-made, *ϕ* = 1.5 mm), boron diamond-doped electrode (Windsor Scientific, *ϕ* = 3 mm), carbon paste electrode, carbon paste electrode doped by cerium(IV) oxide, and carbon paste electrode doped by lanthanum(III) oxide. Carbon paste electrodes were prepared in our laboratory. Also a double-junction reference electrode Ag/AgCl/KCl (3 M) with replaceable outer junction (2.5M KNO_3_) and a platinum wire as an auxiliary electrode were used. The ambient temperature was ca. 23°C. The MTM-Anko EAPro 1.0 software enabled electrochemical measurements, data acquisition, and processing of the results.

Electrochemical impedance spectroscopy measurements were performed using a frequency analyzer (Solartron model FRA 1260) coupled with dielectric interface (model 1296). The surface morphology of electrode material was observed using ultrahigh-resolution scanning electron microscope with field emission (FEG-Schottky emitter; Nova NanoSEM 200, FEI Europe BV) cooperating with EDAX EDS analyzer.

### 2.2. Carbon Paste Electrodes Doped by La_2_O_3_ and CeO_2_

Carbon-based electrodes are useful for voltammetric determination of wide range of analytes in liquid solutions. Moreover, their applicative properties can be improved by doping with metal oxide modifiers. The usage of different doping oxides was reported for modification of carbon-based electrodes so far [18–22]. According to the literature, addition of controlled amounts of cerium(IV) oxide to glassy carbon electrodes material led to the significant enhancement of sensitivity, selectivity, reproducibility, and response time in the amperometric quantification of eugenol [22].

In this work, the experimental data obtained by use of lanthanum oxide-doped graphite paste electrodes applied in voltammetric analysis are presented for the first time. The carbon pastes were prepared by hand mixing an adequate amount of graphite powder and rare earth oxide powder with paraffin oil using a pestle and mortar for at least 30 minutes in the case of each batch. Nanopowders of lanthanum(III) oxide (99.99%) and cerium(IV) oxide (99.9%) were provided by Acros Organics. The ratio of used paraffin oil and graphite powder was determined based on literature repots as well as our experience in order to get electrodes characterized by high chemical and mechanical stability during performance in liquid solutions. After standing overnight, the resulting homogenous pastes were packed into the well of the working electrodes to depth of 2 mm with two different diameters (1.5 and 3 mm). The body of working electrode was a Teflon tube with stainless steel rode of 1.5 mm diameter serving as electric contact. To provide the required smoothness of electrodes, working surfaces the forehead of electrodes were polished on a print paper or tissue paper.

The amounts of used reagents, details of prepared electrodes, and pastes are presented in Table 3.

### 2.3. Chemicals and Glassware

As a supporting electrolyte, buffers of a different pH were prepared in our laboratory (from reagents pure for analysis, POCH, Poland): acetate buffer—mix acetic acid and sodium acetate; Britton–Robinson buffer—mix boric acid, phosphoric acid, acetic acid, and sodium hydroxide; Sørensen phosphate buffer—mix sodium hydrogen phosphate and sodium dihydrogen phosphate; ammonia buffer—mix ammonia and ammonium chloride. As a standard solution, *trans*-anethole (analytical standard, Sigma-Aldrich) was used. 1 *µ*L of solution contains 3.48 *µ*g of *trans*-anethole. Reagents used to determine the impact of interferents are 99% eugenol (Reagent Plus, Sigma-Aldrich), 99% carvacrol (food grade, Sigma-Aldrich), ≥98.5% thymol (pure, Sigma-Aldrich), and zinc, lead, cadmium, bismuth, aluminum, thallium, chromium, and vanadium (all metals from Certipur, Merck). The other chemicals were 95% ethanol (food grade, Polmos, Poland) and 0.1 mol L^−1^ solution of sulfuric acid (pure for analysis, POCH, Poland) for activation of BDD electrode. All reagents used were prepared using quadruply distilled water (two last stages from quartz). Glassware was first immersed in 6 M nitric acid and then rinsed repeatedly with distilled water.

### 2.4. Samples

To verify the possibility to determine anethole in herbal *matrices A*, *B*, and *D*, three solutions were prepared. Also *matrix C* was tested, which did not contain anethole. The composition and preparation of the matrices imitated different anethole-containing beverages. Each matrix was prepared by pouring with the ethanol (95%, food grade) the appropriate herbal composition and the five-day maceration of the mixture. After this time, each matrix was rectified once.

Anethole was also determined in commercially available products such as anise oil (KEJ, Poland), Efe Rakija (Turkey), Ouzo Typnaboy (Greece), and Metaxa^∗^^∗^^∗^ (Greece).

Three independent samples of the same type were tested.

### 2.5. Standard Procedure of Voltammetric Measurements

Measurements were performed using differential pulse voltammetry (DPV). Before each series of measurements, surface of the BDD electrode was activated 15 minutes in 0.1 mol L^−1^ sulfuric acid solution by the potential of 2400 mV. Before each calibration, the BDDE surface was additionally renewed by the potential 1500 mV and time of 30 s in supporting electrolyte. GCE was activated by polishing with polishing powder MicroPolish Alumina 0.05 *μ*m (Buehler, USA).

The investigation of anethole was performed in different supporting electrolytes depending on the working electrode used, that is, 0.1 M acetate buffer with pH 3, 4, 5, or 6; Britton–Robinson buffer with pH 2 and 3; 0.1 M Sørensen's phosphate buffer with pH 6, 7, and 8; or 0.1 M ammonia buffer with pH 9 and distilled water, giving total volume of 5 mL filling the quartz voltammetric cell. The best results were obtained in supporting electrolyte consisting 5 mL of 0.1 M acetate buffer with pH 6. The volume of added standard solution of anethole was of 1–5 *µ*L.

The solution in cell was stirred (ca. 500 rpm) using a magnetic stirring bar. Then, after a rest period of 5 s, differential pulse voltammograms were recorded in the potential window: 0–1200 mV (BDDE), 500–1300 mV (CPE, La_2_O_3_/CPE, CeO_2_/CPE), and 600–1200 mV (GCE). The other standard experimental parameters were as follows: potential step *E*_*s*_ = 5 mV; pulse potential dE = 50 mV; and time of potential step = 40 ms (20 ms waiting time + 20 ms sampling time).

All experiments were performed at 23°C. All experiments were carried out in triplicate.

## 3. Results and Discussion

### 3.1. Carbon Electrodes in Determination of Anethole

**Figure 2:**
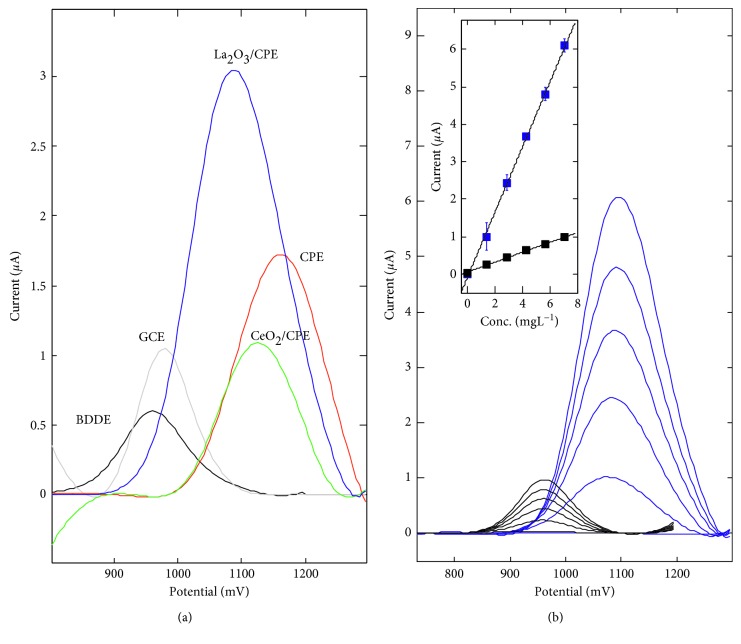
a) DP voltammograms on various carbon electrodes in 0.1 mol L^−1^ acetate buffer of 3.48 mg L^−1^ of anethole; (b) calibration in 0.1 mol L^−1^ acetate buffer of 0, 1.39, 2.78, 4.18, 5.57, and 6.96 mg L^−1^ of anethole on BDDE (black) and La_2_O_3_/CPE (blue).

The purpose of the study was to investigate whether carbon paste electrodes doped by two new rare earth element oxides may be useful in voltammetric determination of the anethole. Commercially available and popular sensors were used as a comparison. The well-defined DPV anethole peak (Figure 2) was obtained on the various carbon electrodes, that is, glassy carbon, boron-doped diamond, carbon paste, and carbon paste doped by lanthanum(III) oxide and cerium(IV) oxide, which were considered in this work. The peak position was observed between 965 and 1155 mV (Table 4, second column). Anodic shift (of 150–200 mV) of anethole oxidation potential has been obtained for carbon paste and two nanoparticles-modified electrodes compared to GCE and BDDE, confirming the lower transfer rate on CPE and nanoparticles/CPE.

**Figure 3:**
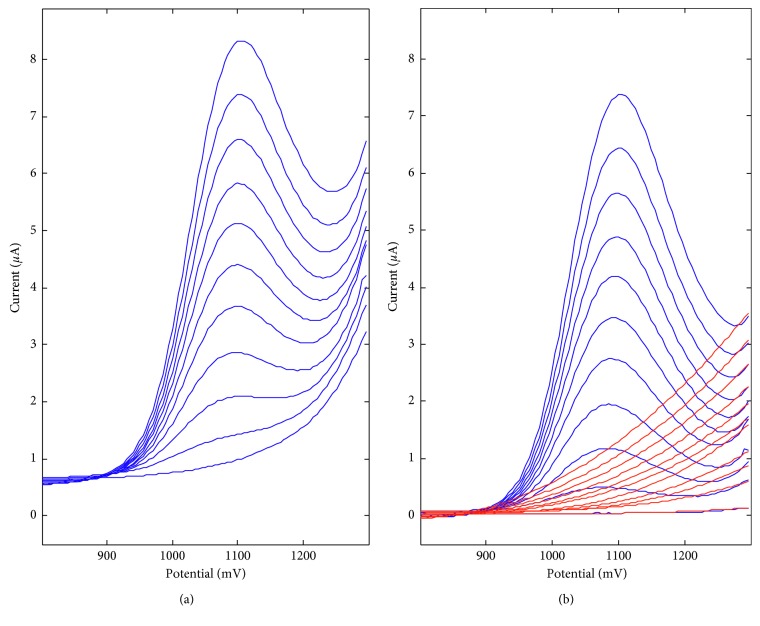
Strategy of baseline interpretation: (a) DP voltammograms in 0.1 mol L^−1^ acetate buffer of 0–6.96 mg L^−1^ of anethole on La_2_O_3_/CPE with experimental baseline; (b) the same voltammograms with subtracted experimental background and simulated baseline by the approximation using 2nd degree polynomials.

Quantitative analysis was preceded by especially projected procedure of baseline modeling and subtraction (Figure 3). The first step of the proposed approach was subtraction of the experimental baseline obtained for the supporting electrolyte. Next, the typical approximation by the polynomial of the 2nd degree was utilized. These two steps were necessary, because the background shape was very different from the polynomial function.

Analytical parameters were determined and tested for two groups of electrodes (Table 4), that is, of the diameter of 3 mm (geometric area of 7.07 mm^2^) and of the diameter of 1.5 mm (geometric area of 1.77 mm^2^). After signal processing, the linear relation between peak current and concentration of the anethole in the range of 0.7–17.5 mg L^−1^ was noticed. Generally, paste electrodes were characterized by the greater sensitivity and lower detection limit in comparison to BDDE and GCE. However, the repeatability of the signal for successive analyte concentration was excellent for the latter (CV < 1%).

The highest sensitivity of 0.89 *µ*A/mg L^−1^ among the considered electrodes was obtained on the carbon paste doped by the 20% of lanthanum(III) oxide nanoparticles, with the correlation coefficient *r* of  0.9993 (for averaged signals for each concentration) and the lowest detection limit of 0.004 mg L^−1^. The sensitivity for the anethole on the electrode doped by the 20% of cerium(IV) oxide nanoparticles (*ϕ* = 3 mm) was even lower (0.34 *µ*A/mg L^−1^) than the reference value obtained on CPE (0.55 *µ*A/mg L^−1^). The lowest sensitivity in the group of sensors with a diameter of 3 mm was obtained on BDDE (0.14 *µ*A/mg L^−1^) what was ca. 6 times less than on La_2_O_3_/CPE. Considering the sensors with a diameter of 1.5 mm, the highest sensitivity of 0.45 *µ*A/mg L^−1^ was obtained on CeO_2_/CPE. For CPE- and La_2_O_3_-doped CPE, the repeatability of the signal relied on the percent (w/w) of the added nanoparticles and was on the level of 5–8% (CV) when the nanopowder addition was lower than 20%. For the addition greater than 20%, the repeatability of the signal rapidly deteriorated (CV > 10%), and therefore these electrodes were not considered in further tests. CP electrodes doped by CeO_2_ did not also show the satisfactory repeatability of the signals recorded for each concentration. It was also observed that increasing addition of the lanthanum(III) oxide nanoparticles decreased sensitivity for the anethole.

For further detailed analysis, La_2_O_3_/CPE (*ϕ* = 3 mm) as a sensor of the greatest sensitivity for the anethole was chosen and for comparison BDDE, which is reliable after appropriate activation. Voltammograms and calibration lines for the anethole in the concentration range from 1.39 to 6.96 mg L^−1^ prepared on the mentioned two sensors are presented in Figure 2.

### 3.2. Supporting Electrolyte Effects

There are several ways in which the supporting electrolytes solvent system can influence mass transfer, the electron reaction (electron transfer), and the chemical reactions which are coupled to the electron transfer. As a supporting electrolyte, 4 different buffers (acetate, Britton–Robinson, phosphate, and ammonia) were applied in examination of the analyte behavior in pH range from 2.0 (BR buffer) to pH 9.0 (ammonia buffer). The best parameters—repeatability, sensitivity, limit of detection, and the favorable relation between signal and baseline—were obtained using acetate buffer; therefore, the pH effect was tested carefully in the pH range typical for this electrolyte, that is, from 3.0 to 6.0. In the considered supporting electrolytes at strongly acidic pH (Britton–Robinson buffer, pH 2.0), neutral pH (phosphate buffer, pH 7.0), and basic pH (phosphate buffer, 8.0; ammonia buffer, pH 9.0), the investigated analyte did not show adequate analytical sensitivity and repeatability.

**Figure 4:**
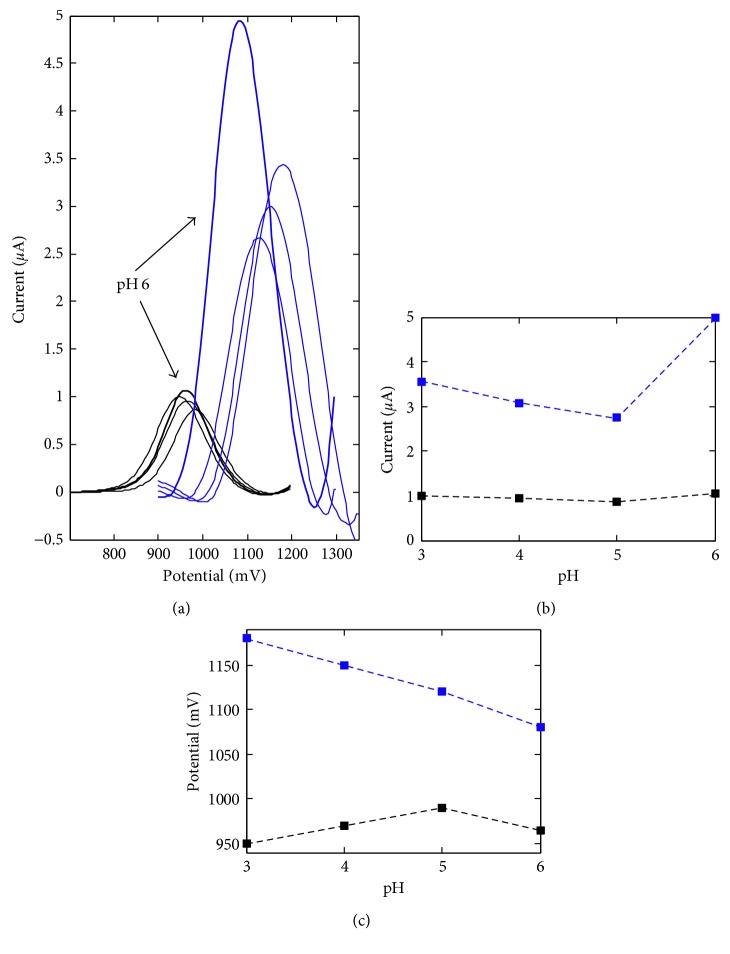
The effect of supporting electrolyte pH on the voltammetric characteristics of anethole on BDDE (black) and La_2_O_3_/CPE (blue): (a) DP voltammograms obtained for 6.96 mg L^−1^ of anethole in 0.1 mol L^−1^ acetate buffer; (b) peak current in relation to pH; (c) peak potential in relation to pH.

Figure 4 presents the influence of the acetate buffer pH on the anethole voltammetric signal. The well-defined DPV peak was observed in the whole range of the considered pH, that is, 3.0–6.0. For BDDE, the peak position changed in the range from 950 to 990 mV, without a distinct maximum current change. For La_2_O_3_/CPE, the oxidation peak currents decreased to pH 5.0 and then increased. Anethole oxidation potential decreased from 1180 mV to 1080 mV as pH increased. Further experiments were done by pH 6, because less positive peak position equal to 1080 mV is more suitable for oxidation. Sensitivity in this case was also ca. 44% greater in comparison with the best variant obtained for the other pH.

### 3.3. Parameters of Anethole Electrooxidation on BDDE and La_2_O_3_/CPE

**Figure 5:**
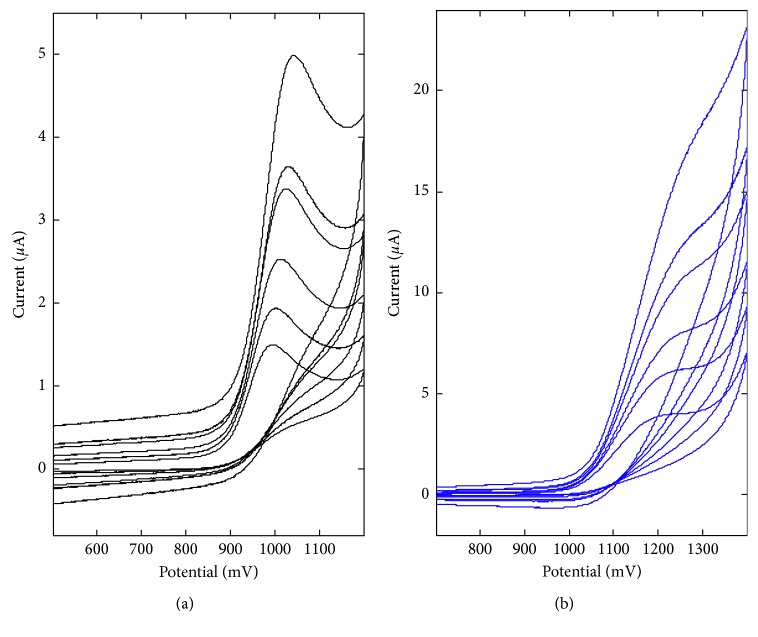
Cyclic voltammograms by the scan rate of 0.025, 0.05, 0.1, 0.2, 0.25, and 0.5 V s^−1^, obtained for 13.92 mg L^−1^ of anethole in 0.1 mol L^−1^ acetate buffer on (a) BDDE and (b) La_2_O_3_/CPE.

The voltammetric behavior of anethole on two carbon electrodes, that is, BDD and La_2_O_3_-modified electrodes, in 0.1 mol L^−1^ acetate buffer of pH 6.0 has been investigated by recording cyclic voltammograms (CV) using the scan rates of 0.025, 0.05, 0.1, 0.2, 0.25, and 0.5 V s^−1^. It was observed that anethole is irreversibly oxidized on these electrodes (Figure 5), what was confirmed by the absence of cathodic step on the backward branch of the CV. The CP electrode modification with La_2_O_3_ nanoparticles leads to the anodic shift of anethole oxidation potential on ca. 200 mV. The effect of potential scan rate in the range of 0.025–0.5 V s^−1^ on the voltammetric behavior of anethole is also presented in Figure 5. The anethole oxidation currents were proportional to the square root of the potential scan rate (1), confirming that the electrochemical process is diffusion controlled [23].

(1) $$\begin{array}{c} \text{BDD}\text{:}i_{p}\left[µ\text{A}\right]=\left(2.59\pm 0.12\right)v^{1/2}\left[{\text{Vs}}^{-1}\right]+\left(0.57\pm 0.05\right),r=0.9953,\\ {\text{La}}_{2}{\text{O}}_{3}\text{/CPE}\text{:}i_{p}\left[µ\text{A}\right]=\left(0.64\pm 0.08\right)v^{1/2}\left[{\text{Vs}}^{-1}\right]+\left(1.34\pm 0.03\right),r=0.9701. \end{array}$$

Moreover, the natural logarithm of anethole peak current (ln*i*_*p*_) increases linearly with the natural logarithm of scan rate (lnν) in the range of potential scan rate under investigation, and the regression equation is described by

(2) $$\begin{array}{c} \text{BDD}\text{:}\ln i_{p}\left[µ\text{A}\right]=\left(0.313\pm 0.003\right)\text{lnv}\left[{\text{Vs}}^{-1}\right]+\left(1.071\pm 0.007\right),r=0.9998,\\ {\text{La}}_{2}{\text{O}}_{3}/\text{CPE}\text{:}\ln i_{p}\left[µ\text{A}\right]=\left(0.073\pm 0.009\right)\text{lnv}\left[{\text{Vs}}^{-1}\right]+\left(0.617\pm 0.021\right),r=0.9700. \end{array}$$

The value of the slope is below the theoretical value of 0.5, what proves the diffusion nature of anethole oxidation peak once again [23]. A linear relationship between the oxidation potential *E_p_* and ln*ν* has been observed, confirming totally irreversible electrode processes:

(3) $$\begin{array}{c} \text{BDD}\text{:}E_{p}\left[\text{V}\right]=\left(0.017\pm 0.001\right)\text{lnv}\left[{\text{Vs}}^{-1}\right]+\left(1.053\pm 0.002\right),r=0.9953,\\ {\text{La}}_{2}{\text{O}}_{3}/\text{CPE}\text{:}E_{p}\left[\text{V}\right]=\left(0.032\pm 0.001\right)\text{lnv}\left[{\text{Vs}}^{-1}\right]+\left(1.301\pm 0.003\right),r=0.9972. \end{array}$$

**Figure 6:**
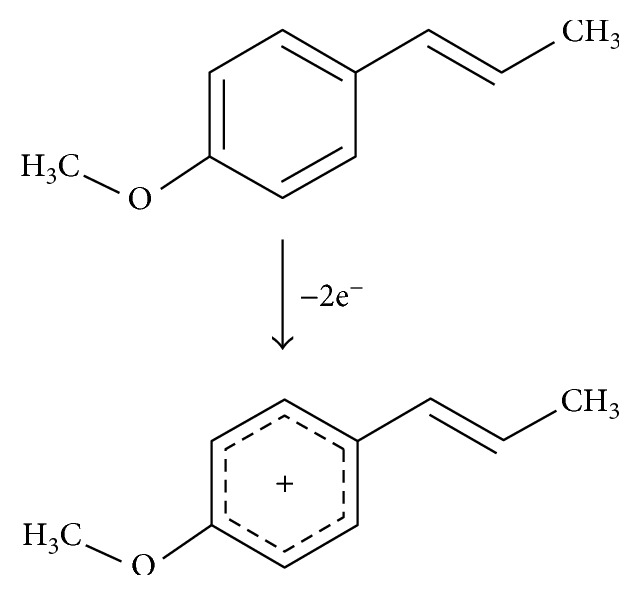
The proposed mechanism of oxidation of *trans*-anethole, on the basis of own experimental data and [25].

In this case, the number of electrons participating in the reaction can be calculated according to [24]

(4) $$\begin{array}{c} E_{p}-E_{p/2}=\frac{47.7}{\left(1-\alpha \right)n_{\alpha }}, \end{array}$$

where *α* is assumed to be 0.5 for a totally irreversible electrode process. The *E*_*p*_ − *E*_*p*/2_ is 53 mV for BDDE and 59 mV for La_2_O_3_/CPE. Hence, the number of electrons participating in the anethole oxidation process equals *n*_*α*_ to 2.22 for BDDE and 2.47 for La_2_O_3_/CPE, what agree well with the values reported earlier [25].  Figure 6 presents the proposition of the electrode reaction.

### 3.4. Investigation by Energy-Dispersive X-Ray Spectroscopy and Scanning Electron Microscopy

**Figure 7:**
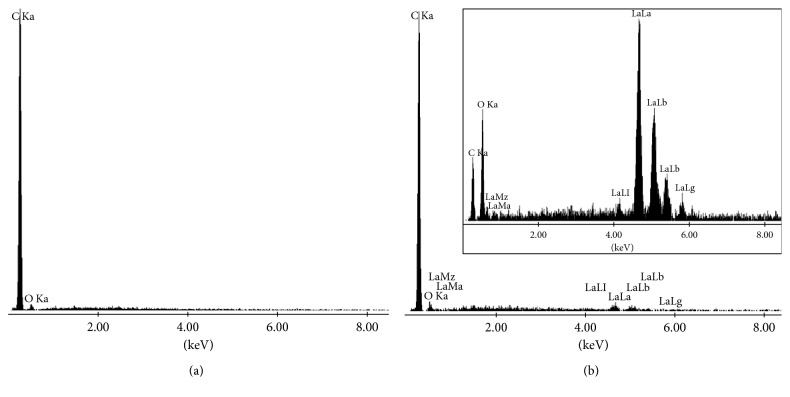
EDX spectrum for (a) nondoped carbon paste; (b) carbon paste doped by lanthanum(III) oxide. Inset: image of lanthanum(III) oxide nanopowder.

The chemical composition of the pastes used for construction of CPE and La_2_O_3_/CPE was analyzed by EDX. The EDX spectrum for the nondoped carbon paste (Figure 7) confirmed the presence of carbon, as the dominant element, and a small amount of oxygen. The EDX spectrum for carbon paste doped by lanthanum(III) oxide (Figure 7) confirmed the presence of the elements carbon, lanthanum, and oxygen.

**Figure 8:**
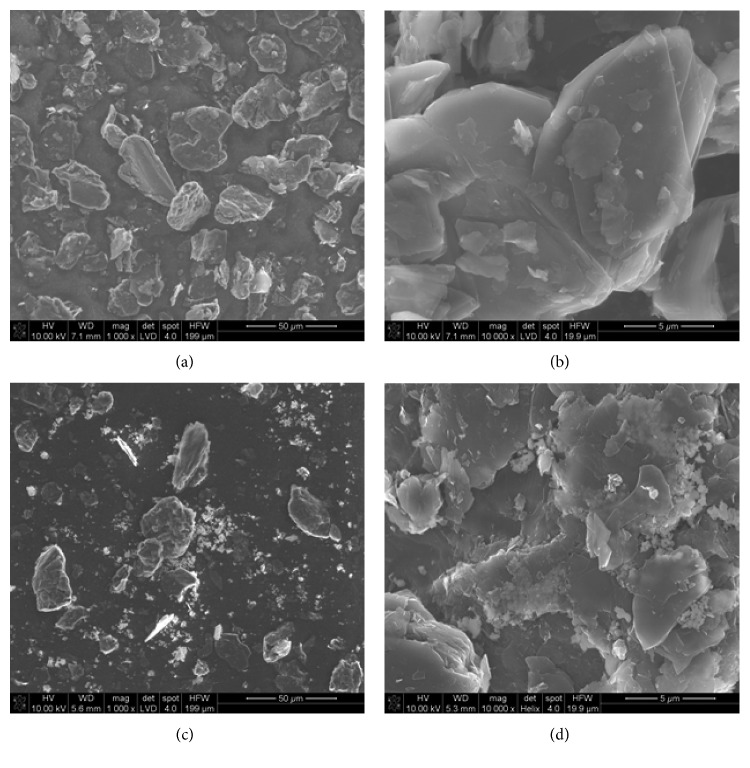
SEM image of the surface: (a) nondoped carbon paste, magnification 1000x; (b) nondoped carbon paste, 10,000x; (c) carbon paste doped by 20% of La_2_O_3_ (w/w), 1000x; (d) carbon paste doped by 20% of La_2_O_3_ (w/w), 10,000x.

The surface morphology of the CPE and La_2_O_3_/CPE was observed by SEM. The SEM test showed that the nondoped carbon paste was characterized by a surface formed by irregular graphite flakes (Figures 8 and 8). The surface of the La_2_O_3_-doped carbon paste is more porous, heterogeneous, and irregular than the surface of nondoped carbon paste (Figures 8 and 8). This suggests that the presence of La_2_O_3_ molecules in carbon paste significantly increases the morphological structure of the material, which facilitates the electron transfer process in the electrode–solution interface, giving better sensitivity and higher repeatability of the voltammetric signal.

### 3.5. Application of Electrochemical Impedance Spectroscopy

Electrical properties of experimental set comprised of the studied carbon paste electrode and Ag/AgCl/KCl reference electrode immersed in solution containing analyte were determined by Electrochemical Impedance Spectroscopy (EIS) method. The measurements were performed in room temperature, with the frequency range of 0.1–10 MHz and the amplitude of sinusoidal voltage signal of 20 mV. The experimental data were analyzed using the ZView software (version 2.2, Scribner Associates, Inc.), which helped in determination of equivalent circuits' optimal parameters.

**Figure 9:**
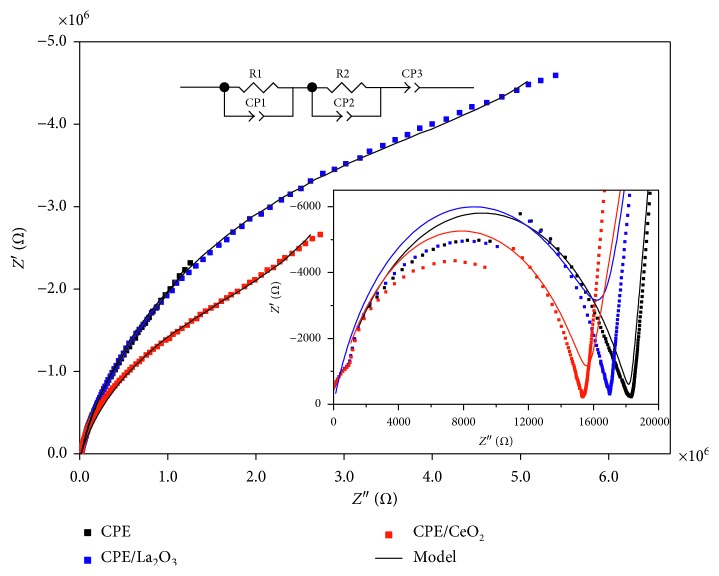
Nyquist's spectra for three different carbon paste electrodes (diameter 3 mm) with fitted equivalent circuit simulations.

The comparison of Nyquist's spectra obtained for three different carbon paste electrodes is given in Figure 9. In each case, spectrum was comprised of semicircle visible in high-frequency range and the spur in middle and low frequencies. Electrical equivalent circuits were fitted to the experimental data sets. Spectra were analyzed by connected in series two parallel equivalent circuits consisted of resistors (R) and constant phase elements (CE) and additional constant phase element indispensable to model the spur in low frequencies. The scheme of the used equivalent model is depicted in inset of Figure 9. The simulated spectra are plotted by solid black line and exhibit good agreement with experimental data presented by points. The semicircle parts of the spectra in high frequencies look similar in each case. The course of above mentioned part of the spectrum depends on the reference electrode and solution used during the measurements. Therefore, the parameters of R1 and CE1 are of similar value (Table 5). The differences in course of spectrum in the middle- and low-frequency parts indicate that it is attributable to carbon paste electrode properties. On the basis of conducted analysis, there is a strong relation between the applicable properties of carbon paste electrode and the value of resistance exhibited in middle-frequency fragments of spectra. In particular, the highest value of resistance R2 shows the electrode modified by lanthanum(III) oxide, while the electrode without rare earth oxide addition is characterized by the lowest value of this parameter. Concomitantly, the lower CE-T-2 value, the better performance of electrode. The most significant differences between behaviors of studied electrodes are visible in low frequencies part of the spectra. It is reflected in particular in CE3 element. CE-T-3 value determined for undoped electrode is of order higher than for electrodes modified by rare earth metal oxides. Moreover, parameter *n*3 for CPE is somehow higher than for doped electrodes and close to 1, indicating stronger capacitive properties of undoped graphite than in the case of electrodes modified modified by lanthanum and cerium oxides.

### 3.6. Interferences

Such parameters as potential window, potential step, potential pulse, and time of potential step were tested to optimize the procedure of the anethole determination. The criteria of optimization were repeatability, sensitivity of the method, and the favorable relation between signal and baseline. It was observed that starting potential does not have influence on the anethole peak. Taking into account all the criteria selected experiment conditions are potential step 5 mV, potential pulse 50 mV, and time of the potential step 40 ms (i.e., waiting time 20 ms + sampling time 20 ms).

As possible interferences, metal ions such as Zn(II), Pb(II), Cd(II), V(III), Bi(III), Al(III), Tl(I), and Cr(III) and organic compounds such as eugenol, carvacrol, and thymol were tested, which may be present in plants and products of biological origin, in which anethole also occurs. The concentration of the metal ions was in the range of 1.4–14 mg L^−1^ by the 13.92 mg L^−1^ of anethole, which was present in the measured solution. The anethole peak position was not moved, and also no additional peaks were observed. However, the impact of the analyzed ions on the height of the anethole peak after addition of metals was noticeable: change for BDDE anethole signal was in the range of 93–99%, and change for La_2_O_3_/CPE was in the range from 82 to 127% (Table 6). The greater sensitivity variation in the last case may be connected with the chemical reactions between metal ions and active lanthanum(III) oxide nanoparticles, what could cause the change of the number of active centres on the electrode surface.

No additional current peaks coming from eugenol, carvacrol, and thymol were observed in the considered acetate buffer (pH 6.0) and potential area where anethole peak was recorded. The concentration of the added substances was in the range of 0.7–3.5 mg L^−1^ by the 13.92 mg L^−1^ of anethole. The presence of biological compounds in the solution caused, in the experiments with BDDE, increase of the sensitivity up to 100%. This value is related to the study of the carvacrol effect at the 4 times excess of anethole. The mentioned interferents may facilitate the charge transfer between the analyte and the electrode. In the case of measurements on La_2_O_3_/CPE, an addition of 3 biological compounds resulted in the change of the signal amplitude in the range of 84–126%.

### 3.7. Determination of Anethole in Herbal Matrices and Commercial Products

**Figure 10:**
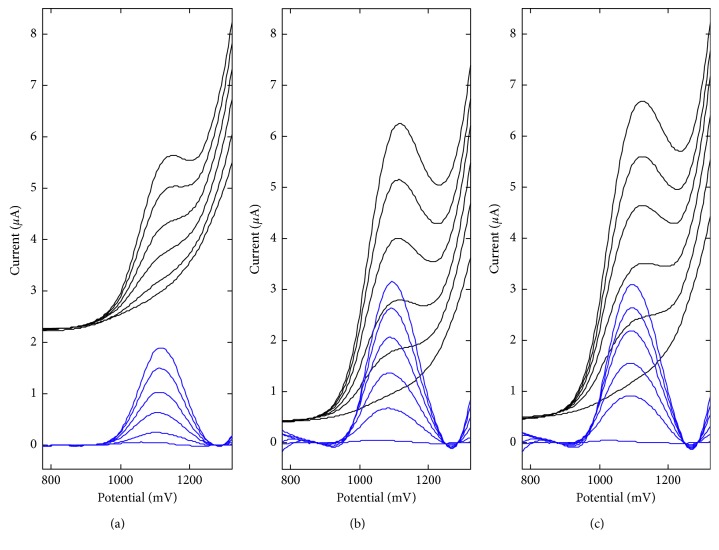
Standard addition voltammograms (black) and the same curves after subtracting experimental background, and next simulated baseline approximated using 3rd degree polynomials (blue), in determination of the anethole in 0.1 mol L^−1^ acetate buffer on La_2_O_3_/CPE, in (a) *matrix D*, (b) Ouzo, and (c) Raki. Each voltammogram set: background, sample, and 1.39, 2.78, 4.18, and 5.57 mg L^−1^ standard additions of anethole.

Because anethole occurs in food products (beverages, herbal oils, and tinctures) and herbs such as anise, star anise, fennel, liquorice, and caraway, the problem of anethole determination of specially prepared herbal matrices was considered. The composition of these mixtures which mimics the real commercially available products is given in Table 7. It is important that some matrices contain anethole, while the other did not contain this analyte, and it was added at the stage of recovery studies. The concentration of the anethole in *matrices A*, *B*, and *D* was on the level 0.1–1.6 g L^−1^ (Table 7). The highest concentration was in the most complex mixture *B*, while the lowest in *D*, where only one component contained anethole. The significant decrease of the sensitivity of the method was observed in comparison to the measurements in only supporting electrolyte. The decrease was to 73% (*matrix B*) in the case of BDDE and to 66% (*matrix D*) in the case of La_2_O_3_/CPE. Exemplary voltammograms recorded on La_2_O_3_/CPE in the case of anethole determination in *matrix D* are presented in Figure 10.

*Matrix C* did not contain a detectable concentration of the anethole; therefore, this analyte was added to the herbal extract, and percent of recovery was studied (Table 8). Using BDDE, the concentration of anethole of 3.5–10.5 mg L^−1^ was successfully determined with recovery 101–108%. La_2_O_3_/CPE enabled determination of 1.4–4.2 mg L^−1^ of anethole with recovery 95–100%. The correlation coefficient *r* in each case was greater than 0.995. The presence of herbal *matrix C* caused a significant decrease of the method sensitivity, that is, ca. 30% in the case of BDDE and even 50% in the case of La_2_O_3_/CPE.

Further, anethole was determined in commercially available products. Some research objects were chosen in which the mentioned analyte is present on very low and very high concentration level. The measurements were done without sample preparation or anethole extraction. The adequate sample volume was added directly to the electrochemical cell. It was observed (Tables 9 and 10) that, in anise essential oil, the concentration of anethole was ca. 570 g L^−1^, while in alcohol drinks, like Metaxa, Ouzo, and Rakija, it was ca. 0.13–0.21 g L^−1^. The results obtained using both electrodes were compatible. At 95% confidence level, the calculated Student's *t*-values for the replicate measurements of each sample (Table 10) using both fabricated sensors did not exceed the theoretical value (2.7765), indicating that the results obtained are not significantly different. An *F*-test revealed no significant difference between the standard deviations of the two sets of replicate measurements for each sample. Exemplary voltammograms recorded on La_2_O_3_/CPE in the case of anethole determination in Ouzo and Raki are presented in Figures 10 and 10.

## 4. Conclusions

In this work, a sensitive, rapid, and convenient DPV procedure of anethole determination was proposed, which does not require sample preparation and separation of the analyte, even in the case of complex matrices. Additionally, it was proved that various carbon electrodes, that is, BDD, GC, CP, and CP doped by La_2_O_3_, and CeO_2_ nanoparticles, are sensitive for anethole, and the proposed analytical strategies fulfill typical validation criteria. Recording cyclic voltammograms, it was noticed that electrooxidation process has totally irreversible character, controlled by diffusion, in which two electrons take part.

The most sensitive electrode turned out to be La_2_O_3_/CPE with 20% of nanoparticles in graphite paste (w/w). According to our knowledge, it is the first literature report about application of such a sensor. Sensitivity obtained in DPV experiments realized by optimized parameters in 0.1 mol L^−1^ acetate buffer was for La_2_O_3_/CPE of 3 mm diameter equal to 0.885 ± 0.016 *µ*A/mg L^−1^ with the correlation coefficient *r* of 0.9993 and the detection limit of 0.004 mg L^−1^, while for commercially available sensor BDDE it was 0.135 ± 0.003 *µ*A/mg L^−1^ with *r* of 0.9990.

Operation of the selected electrodes was verified using especially prepared herbal ethanol extracts which contained and did not contain anethole. In the last case, recovery was tested applying standard addition method. Anethole was also successfully determined in commercially available products, such as anise essential oil, which contains a large concentration of anethole, and in alcohol drinks like Metaxa, Ouzo, and Rakija, in which the considered analyte occurs on trace levels. The results obtained on La_2_O_3_/CPE and BDDE were statistically consistent, at 95% confidence level.

## Figures and Tables

**Table 1:** Content of anethole in various essential oils.

Type of plants	Country of origin	Part of plants	Method of obtaining oil and analysis	Content of anethole in the oil (%)	Reference
Fennel	Brazil	Seed	Supercritical fluid extraction (SFE), GC-MS	11.0–47.4-*trans*	[1]
Star anise	India	Fruit	Distillation in Clevenger-type apparatus, GC-MS	1.07-*cis*	[7]
				75.62-*trans*	
Anise	Germany	Seed	Distillation in Clevenger-type apparatus, GC-MS	0.14-*cis*	[8]
				82.10-*trans*	
Caraway	Different European countries	Fruit	Steam-distillation, GC-FID	0–2.2-*trans*	[9]
Bitter orange	Algeria	Peel	Distillation in Clevenger-type apparatus, GC-MS	2.3-*trans*	[10]

**Table 2:** Selected methods for quantification of both isomers of anethole in various matrices.

Type of matrix	Analytical technique	Linearity range (mg L^−1^)	LOD (mg L^−1^)	LOQ (mg L^−1^)	*Trans*-anethole conc. (mg L^−1^)	Reference
Fennel essential oil	HPLC	10–100	0.95	3.0	0.2–30.5	[15]
Fennel essential oil	GC-MS	10–550	0.002	0.006	0.16–40	[15]
Aniseed drinks	HPLC	2–16	0.0023	0.0077	125–4040	[16]
Human blood serum	HS-SPME-GC-MS	0.002–0.2	0.0036	0.0053	0.0054–0.0176	[17]

**Table 3:** Details of paste preparation and CP electrodes parameters.

Electrode	Electrode diameter (mm)	Ingredients	Paste label	Weight ratio of powder ingredients
CeO_2_/CPE 20%	1.5	2 g graphite, 1.5 ml paraffin oil, 0.5 g CeO_2_	PCe20-5	0.2 CeO_2_–0.8 graphite
	3			
La_2_O_3_/CPE 20%	1.5	2 g graphite, 1.5 ml paraffin oil, 0.5 g La_2_O_3_	PLa20-2	0.2 La_2_O_3_–0.8 graphite
	3			
La_2_O_3_/CPE 30%	1.5	2 g graphite, 1.5 ml paraffin oil, 0.857 g La_2_O_3_	PLa30-3	0.3 La_2_O_3_–0.7 graphite
	3			
La_2_O_3_/CPE 40%	1.5	2 g graphite, 1 ml paraffin oil, 1.333 g La_2_O_3_	PLa40-4	0.4 La_2_O_3_–0.6 graphite
	3			

**Table 4:** DPV anethole determination in the range of 0.7–17.5 mg L^−1^ in 0.1 mol L^−1^ acetate buffer, pH 6.0 (*n* = 3).

Electrode	Anethole peak position (mV)	*a* ± SD_*a*_ (*µ*A/mg L^−1^)	*b* ± SD_*b*_ (*µ*A)	*r*	LOD (mg L^−1^)
*Electrode diameter 3 mm, area 7.07 mm* ^*2*^					
BDDE	965	0.135 ± 0.003	0.050 ± 0.013	0.9990	0.024
GCE	990	0.306 ± 0.005	0.031 ± 0.046	0.9995	0.011
CPE	1155	0.546 ± 0.036	−0.068 ± 0.083	0.9936	0.006
CeO_2_/CPE 20%	1125	0.341 ± 0.009	−0.060 ± 0.021	0.9989	0.010
La_2_O_3_/CPE 20%	1095	0.885 ± 0.016	0.076 ± 0.069	0.9993	0.004
*Electrode diameter 1.5 mm, area 1.77 mm* ^*2*^					
GCE	970	0.022 ± 0.001	0.0035 ± 0.0051	0.9989	0.148
CPE	1110	0.111 ± 0.004	−0.005 ± 0.043	0.9983	0.030
CeO_2_/CPE 20%	1075	0.227 ± 0.008	−0.054 ± 0.080	0.9978	0.014
La_2_O_3_/CPE 20%	1080	0.449 ± 0.021	−0.058 ± 0.044	0.9959	0.007
La_2_O_3_/CPE 30%	1095	0.350 ± 0.016	−0.039 ± 0.035	0.9958	0.009
La_2_O_3_/CPE 40%	1100	0.332 ± 0.010	0.029 ± 0.110	0.9980	0.010

**Table 5:** Parameter values of fitted equivalent circuits from Figure 9.

Frequency range	Parameter	Electrode		
		CPE	CeO_2_/CPE	La_2_O_3_/CPE
High (10^6^–10^7^ Hz)	R1/Ω	18,248	15,680	16,751
	CE-T-1/Ss^n^	7.850 × 10^−10^	6.262 × 10^−10^	5.009 × 10^−10^
	*n*1	0.720	0.750	0.777
Medium (10^6^–10^2^ Hz)	R2/Ω	1.857 × 10^6^	2.272 × 10^6^	6.027 × 10^6^
	CE-T-2/Ss^n^	7.05 × 10^−7^	2.65 × 10^−7^	1.21 × 10^−7^
	*n*2	0.875	0.841	0.857
Low (10^2^–10^−1^ Hz)	CE-T-3/Ss^n^	1.03 × 10^−6^	6.66 × 10^−7^	5.81 × 10^−7^
	*n*3	0.940	0.765	0.881

**Table 6:** Influence of interferences (*n* = 3) on anethole peak of 13.92 mg L^−1^ in 0.1 mol L^−1^ acetate buffer, pH = 6.0.

Interferent	Proportion anethole: interferent	*i*_anethole+interferent_/*i*_anethole_	
		BDDE	La_2_O_3_/CPE 20%
*Metal ions*			
Zn^2+^	20 : 0	1.00	1.00
	20 : 2	0.99	1.02
	20 : 10	0.99	1.21
	20 : 20	0.99	1.27
Cd^2+^	20 : 0	1.00	1.00
	20 : 2	0.99	1.07
	20 : 10	0.98	1.12
	20 : 20	0.96	1.15
Pb^2+^	20 : 0	1.00	1.00
	20 : 2	0.99	1.04
	20 : 10	0.99	1.10
	20 : 20	0.98	1.13
V^3+^	20 : 0	1.00	1.00
	20 : 2	1.00	0.95
	20 : 10	0.99	0.88
	20 : 20	0.99	0.82
Bi^3+^	20 : 0	1.00	1.00
	20 : 2	0.99	1.02
	20 : 10	0.98	1.04
	20 : 20	0.96	1.22
Al^3+^	20 : 0	1.00	1.00
	20 : 2	1.00	1.02
	20 : 10	0.99	1.02
	20 : 20	0.99	1.03
Tl^+^	20 : 0	1.00	1.00
	20 : 2	0.98	0.99
	20 : 10	0.95	0.91
	20 : 20	0.93	0.90
Cr^3+^	20 : 0	1.00	1.00
	20 : 2	0.99	1.12
	20 : 10	0.99	1.22
	20 : 20	0.95	1.23
*Organic compounds*			
Eugenol	20 : 0	1.00	1.00
	20 : 1	1.19	1.14
	20 : 2	1.46	1.14
	20 : 5	1.90	0.84
Thymol	20 : 0	1.00	1.00
	20 : 1	1.11	1.11
	20 : 2	1.16	1.21
	20 : 5	1.32	1.26
Carvacrol	20 : 0	1.00	1.00
	20 : 1	1.23	1.09
	20 : 2	1.55	1.07
	20 : 5	2.00	1.04

**Table 7:** Determination of anethole in four herbal matrices (*n* = 3).

Matrix label	Composition of herbal mixture		Amount of ethanol for maceration	In the matrix may be anethole	Anethole conc. ± SD (g L^−1^)
*A*	*Fruit of anise*	7.5 g	25 mL	Yes	BDDE: 0.143 ± 0.005 La_2_O_3_/CPE: 0.102 ± 0.024
	*Fruit of star anise*	2.5 g			
*B*	Hyssop leaves	2.125 g	50 mL	Yes	BDDE: 1.63 ± 0.07 La_2_O_3_/CPE: 1.61 ± 0.19
	Root of the sweet flag	0.45 g			
	Lemon balm leaves	1.5 g			
	*Fruit of anise*	7.5 g			
	*Fruit of star anise*	2.5 g			
	*Fruit of fennel*	6.25 g			
	Fruit of coriander	0.75 g			
*C*	Wormwood leaves	7.5 g	50 mL	No	BDDE < LOD La_2_O_3_/CPE < LOD
*D*	Wormwood leaves	7.5 g	50 mL	Yes	BDDE: 0.500 ± 0.005 La_2_O_3_/CPE: 0.475 ± 0.033
	Hyssop leaves	2.125 g			
	Root of the sweet flag	0.45 g			
	Lemon balm leaves	1.5 g			
	*Fruit of fennel*	6.25 g			
	Fruit of coriander	0.75 g			

Italic signs herbs from which it is possible to extract anethole.

**Table 8:** Standard addition voltammetry of anethole in herbal matrix *C* (n = 3).

Electrode	Added (mg L^−1^)	*a* ± SD_*a*_ (*µ*A/mg L^−1^)	*b* ± SD_*b*_ (*µ*A)	Found ± SD (mg L^−1^)	Recovery (%)	*r*
BDDE 3 mm	3.48	0.0939 ± 0.04	0.3460 ± 0.05	3.68 ± 0.70	105.87	0.9951
	6.96	0.0973 ± 0.03	0.6859 ± 0.03	7.05 ± 0.51	101.30	0.9982
	10.44	0.0963 ± 0.04	1.0814 ± 0.02	11.22 ± 0.61	107.51	0.9987
La_2_O_3_/CPE 3 mm	1.39	0.4306 ± 0.02	0.5995 ± 0.11	1.39 ± 0.19	100.02	0.9951
	2.78	0.435 ± 0.003	1.186 ± 0.001	2.728 ± 0.039	95.79	0.9999
	4.18	0.4409 ± 0.01	1.8124 ± 0.01	4.11 ± 0.07	98.45	0.9999

**Table 9:** Determination of anethole in commercial products, calibration parameters (*n* = 3).

Samples	*a* ± SD_*a*_ (*µ*A/mg L^−1^)	*b* ± SD_*b*_ (*µ*A)	*r*
*BDDE, electrode diameter 3 mm, area 7.07 mm* ^*2*^			
Anise essential oil	0.0293 ± 0.001	0.0667 ± 0.005	0.9967
Metaxa^∗^^∗^^∗^	0.0535 ± 0.002	0.0691 ± 0.008	0.9975
Ouzo	0.0905 ± 0.004	0.1699 ± 0.014	0.9971
Raki	0.1068 ± 0.004	0.2216 ± 0.018	0.9967
*La*_*2*_ *O*_*3*_ */CPE, electrode diameter 3 mm, area 7.07 mm* ^*2*^			
Anise essential oil	0.3682 ± 0.018	0.8544 ± 0.078	0.9951
Metaxa^∗^^∗^^∗^	0.8061 ± 0.032	1.1490 ± 0.068	0.9968
Ouzo	0.7177 ± 0.020	1.4659 ± 0.067	0.9989
Raki	0.4497 ± 0.010	0.9507 ± 0.034	0.9993

**Table 10:** Anethole concentration in commercial products (*n* = 3).

Samples	Anethole conc. ± SD/g L^−1^		*F*-test	*t*-test
	BDDE	La_2_O_3_/CPE		
Anise essential oil	569.0 ± 62.5	580.00 ± 77.5	1.5376	0.1914
Metaxa^∗^^∗^^∗^	0.129 ± 0.019	0.143 ± 0.014	1.8420	1.0275
Ouzo	0.188 ± 0.022	0.204 ± 0.014	2.4691	1.0627
Raki	0.208 ± 0.025	0.211 ± 0.012	4.3403	0.1874
